# Implied Consent for HIV testing in the United Kingdom: time for a new approach?

**DOI:** 10.1016/S2352-3018(21)00276-9

**Published:** 2021-12-08

**Authors:** David Chadwick, Emma Page, Dominic Wilkinson, Julian Savulescu

**Affiliations:** 1 Centre for Clinical Infection, James Cook University Hospital, Middlesbrough TS4 3BW, UK; 2 Department of Virology, Leeds Teaching Hospitals NHS Trust, Leeds LS1 3EX, UK; 3 The Oxford Uehiro Centre for Practical Ethics, University of Oxford, Littlegate House, St Ebbes Street, Oxford OX1 1PT, UK

**Keywords:** HIV, consent, testing, AIDS, exceptionalism

## Abstract

Despite HIV infection being a treatable chronic illness and with many advances in testing for HIV, late diagnosis is still common with associated avoidable morbidity and mortality. Requirements for explicit consent for HIV testing are different to other blood tests and are major barriers to testing. We argue that the disparity is illogical and outdated. We propose a model for normalising HIV testing which allows routine testing in various healthcare settings via implied consent, where other blood tests are being performed. Inclusion of testing for hepatitis B and C might also be incorporated into this model. The ethical argument for this is principally beneficence towards those with undiagnosed infection and those they may infect. Patient autonomy would be maintained using systems allowing individuals to opt-out of implied consent.

## Introduction

Four years ago a 53 year old white woman was admitted to a hospital in England with severe *Pneumocystis jiroveci* pneumonia and a new diagnosis of advanced HIV infection. Despite intensive care, she died 10 days later. A review of her interactions with primary and secondary care over the previous 5 years identified four separate episodes when an HIV test should have been offered according to national guidance, including recurrent shingles and a glandular fever-like illness. Had she been tested and started antiretroviral therapy, it is very likely she would still be alive.

Since the late 1990s HIV has been a treatable infection. However over 40% of people with a new diagnosis of HIV in the UK still present late (CD4 count < 350/mm^3^).^1^ Late HIV diagnosis is the most important predictor of morbidity and mortality: people diagnosed late have a 10-fold increased risk of death within a year of diagnosis.^2^ Late diagnosis also perpetuates spread of HIV since antiretroviral therapy largely eliminate transmission.

## HIV exceptionalism and testing

One of the main drivers of late HIV diagnosis is failure to test for HIV. Missed opportunities to test for HIV are common in the years prior to diagnosis.^3,4^ However traditionally testing for HIV has been regarded as different to virtually all other blood tests, requiring pre-test counselling and explicit consent. This has been called ‘HIV exceptionalism’.^5^ Consequently testing for HIV has been more arduous, requiring explicit consent (in some jurisdictions in written form).

In the UK, the General Medical Council (GMC) has previously given specific advice around testing for HIV, stating that explicit consent should be obtained. In the 1980s and 1990s, this was appropriate as a positive HIV test was similar to a positive genetic test for Huntington’s Disease in terms of poor prognosis with no effective treatment.

With the advent of highly active antiretroviral therapy (HAART) in 1996, outcomes improved.^6^ This led, in 2006, to calls to reduce barriers to testing.^7^ Furthermore, advances in diagnostic technology have provided additional options for testing such as rapid, point-of-care tests including self-testing kits. However the GMC has not updated UK guidance for HIV testing, instead providing more general guidance around consent and investigations, with a proportionate approach advocated, recognising that for many routine blood tests verbal consent is adequate. However, the guidance still insists that clinicians should give patients information about tests being performed.

One approach to promote HIV testing, sometimes called ‘opt-out’ testing, has been discussed for over a decade. This is where pre-test counselling is not required, and the clinician discloses that an HIV test is planned, usually in addition to other blood tests, with the patient able to opt out (decline). In 2006, the CDC revised its HIV testing guidance in active support of opt-out testing^9^. The positive impact of opt-out testing versus consent-in (opt-in) testing on HIV testing uptake has been demonstrated in randomised controlled trials.^10^

Whilst this approach has probably (but not always) led to increased testing, more needs to be done. In the United States around 13% of people living with HIV are undiagnosed,^11^ while in the UK it is 6%.^1^ This suggests that opt-out testing at traditional testing sites such as sexual health clinics and antenatal booking appointments may not be adequate, and expanded testing in non-traditional sites such as Emergency Departments (ED) is required.^12^

Current guidance in most countries (including that from the British HIV Association^13^) continues to state that the patient should be aware that an HIV test is being done and that for every individual tested, ‘basic information given should include how results can be accessed, the advantages of testing, availability and effectiveness of treatments, prevention and the window period’. This requires considerable resources, particularly in busy front-line settings where it may not be possible to engage in the necessary counselling, information provision and giving test results. This has perpetuated the hesitancy or inability of clinicians to test more widely.

In contrast, for many blood tests, for example biochemistry or blood counts, discussions about precisely what the tests are looking for is rare. For routine blood tests, the concept of ‘presumed’ or ‘implied’ consent is generally assumed: that by allowing someone to take blood from their arm, the patient is effectively consenting to such tests despite no discussion of implications of abnormal results. One of the UK HIV Commission’s major recommendations was to make HIV testing a routine procedure: ‘Every blood test undertaken that isn’t also used as a chance to test for undiagnosed HIV, is an opportunity missed’.^14^

There is a spectrum of information provision and consent that applies to diagnostic tests in medicine (Table 1). Our choices about how much information to provide, and how to verify the patient’s consent depend on our ethical evaluation of the reasons to test, and the risks or harms of testing.

## Reasons for changing requirements for consent

There are three broad reasons to consider revising the current approach to HIV testing. The first is on the basis of consistency. The second is based on the benefit to the patient of testing. The third is based on the benefit to others of testing.

One important question is why HIV testing is treated any differently from other tests? It can’t be because of the seriousness of disease potentially detected. Routine tests, usually involving limited/no information provision and implied consent, may identify pathologies with a worse prognosis than HIV such as acute leukaemias or renal failure. For consistency, we ought to take a similar informed consent approach to HIV as for other chronic illness.

Because HIV is now a treatable chronic illness, whose treatment is most effective when started earlier, most patients would presumably wish to know their diagnosis at the earliest opportunity. One key feature of identifying undiagnosed HIV infection (compared with renal failure or leukaemia) is that the benefit is not just to the individual tested, but also to others who won’t be infected once that individual is aware of their HIV status and virologically suppressed on treatment. It would therefore be both in an individual’s best interest to know and in the wider society’s interest in terms of transmission of HIV.

If the benefit to others is significant, there can be an ethical argument for mandatory testing for communicable diseases. Such arguments have been made recently in the COVID-19 pandemic with some governments implementing mandatory COVID-19 testing and vaccination.^15^ There is a further argument for expanded testing based on reducing costs to the health system. Most studies evaluating expanded HIV testing have identified new infections at rates above the 1/1,000 threshold of cost-effectiveness, implying cost savings if universally applied.^16,17^

## Counterarguments

There are two arguments against changing the status quo. The first is that patient autonomy requires informed decision making, as described in the GMC guidance. There has been strong opposition to proposals for mandatory COVID-19 testing outside international travel. Likewise, requiring less explicit consent for HIV testing, for example by reducing the threshold for implied consent, risks compromising patient autonomy. Perhaps any problems with testing for HIV should be addressed by health services simply trying harder to test while obtaining consent properly? Furthermore, some might contend that patients should receive detailed information and agree to all forms of medical tests.

In an ideal world this argument would be persuasive. However the reality of many health services is that most interactions with patients are short due to service pressures and routine conversations around HIV (or other) testing are not feasible. While implementation of opt-out HIV testing strategies in front-line settings is sometimes possible, sustainability is the main challenge due to the perpetual training needs of high turnover, frontline staff with limited resources. Thus alternatives to opt-out testing need to be considered.

The second response might draw on the views of patients about what they would wish to know. Much evidence from those tested for HIV in the 1980s without their knowledge, after contaminated blood transfusions, showed the hurt they felt as no consent was sought nor discussion of the implications of a positive test. This is understandable given their diagnosis was in an era when there was no effective treatment, more stigma associated with the infection and many were not informed of their diagnosis. Nonetheless, despite stigma still being present, recent surveys of patients exploring normalisation of testing and potential change of consent processes have shown a surprising willingness to remove the exceptionalism still associated with testing.^18,19^ Of course, it is essential that patients be informed of their results and follow up implemented, to facilitate better future health care and promote their autonomy. Post-test counselling, not pre-test counselling, is most important.

## Expanding screening for HIV

There is a strong ethical case for expanding the current approach to HIV testing. One approach would be to include HIV testing routinely on blood samples collected for reasons other than a suspicion of HIV infection. This approach would seek to normalise HIV testing when any blood test is ordered in a similar way to the normalisation of SARS-CoV-2 nasopharyngeal tests recently. Aside from HIV, hepatitis B and C might be routinely incorporated into a test ‘bundle’. This would provide an opportunity to identify individuals with undiagnosed HIV infection or those lost to regular HIV care. In this situation, since blood is already being drawn, it constitutes no inconvenience to the patient and might be covered by existing implied consent.

A concept similar to this (previously described as ‘notional consent’ for BBV testing), has emerged and been used in some UK EDs. In this scenario, to minimise operational barriers to testing in busy clinical settings, patients undergoing blood tests are made aware they will be tested for BBVs via posters or leaflets in waiting areas and blood testing, with the opportunity to opt out. This approach has been pioneered in several EDs where the local prevalence of HIV is greater than 1/1,000, the threshold where universal testing is cost-effective, with impressive results in terms of identifying undiagnosed infections.^18,20^ In one such study high testing rates were maintained for a 19 month period. A frequently voiced obstacle to implementation of testing in non-traditional settings is clinical ownership of positive results. EDs cannot be expected to take on this role. Electronic reporting all positive results directly to the relevant specialist teams is an alternative approach that has been successfully implemented.^18^

This approach could be expanded beyond EDs to all situations where patients have blood tests. Test request systems could avoid frequent repeat testing to ensure the tests remain cost-effective. Information would be made available to patients undergoing blood tests that a panel of infections will be periodically tested unless they opt out, along with information on the employment and insurance implications of testing positive. Following a public health information campaign, it would be possible for people to opt out either at the point of having blood tests, or via a national register. This would not require specific discussion of BBV screening each time blood is drawn. Crucially, detailed, sensitive, confidential and informative counselling should occur for any patients who test positive.

The advantage of a community wide screening programme for HIV and other BBVs is that it should dramatically reduce late BBV diagnoses. It would protect both those with undiagnosed infection and their partners and significantly reduce the rate of community transmission of these serious infections. A similar system currently operates for organ donation in England, Wales and Scotland, since an opt-out approach to consent for donation was introduced. Clearly any forum to explore this proposal would need the involvement of a wide range of public and patient representatives to determine the best way to make it feasible and acceptable.

## Conclusion

The exceptionalism around HIV testing and consent is no longer justified given the continuing high levels of undiagnosed infections, late diagnosis and advances in treatment. Given the overwhelming benefits of identifying those with undiagnosed infections for that individual and to the wider community, there are valid ethical arguments to treat this test at least similarly to other treatable chronic infections and explore other ways to normalise and expand testing. Developing a framework to do this, including patients, policy makers and health professionals is therefore, we believe, a priority.

## Figures and Tables

**Table 1:** Spectrum of consent for tests

Information provision	Consent
A. **No**/minimal **information provided** unless requested e.g. many forms of screening tests, tests required by law	i. Implied consent (most hospital tests) or Compulsory (testing even if refusal) if legally required e.g. alcohol testing after a motor vehicle accident
B. **Information provided at population level** e.g. NHS Health Check	ii. Default testing with opt-out (testing unless previously opted out) Expanded HIV testing?*
C. **Information provided at local level (e.g. posters/leaflets)** e.g. some screening tests for cancer	iii. Implied (testing if patient’s body language implies agreement to test) e.g. most outpatient blood tests Expanded HIV testing?*
D. **Brief information at time of test** e.g. some screening tests for cancer	iv. Verbal (patient indicates willingness to test) e.g. screening “combined test” during pregnancy *Opt-out HIV testing (current norm)*
E. **Detailed information at time of test** e.g. some forms of genetic testing	v. Written e.g. some forms of genetic testing *Traditional HIV testing (consent-in) with pre-test counselling*

The spectrum of informed consent for diagnostic tests. *Potential ethical consent model for expanded HIV testing.
